# The Roles of Serotonin in Decision-making under Social Group Conditions

**DOI:** 10.1038/s41598-018-29055-9

**Published:** 2018-07-16

**Authors:** Young-A Lee, Yukiori Goto

**Affiliations:** 1 Department of Food Science and Nutrition, Daegu Catholic University, Gyeongsan, Gyeong-buk 38430 South Korea; 2 Primate Research Institute, Kyoto University, Inuyama, Aichi 484-8506 Japan

## Abstract

People in a social group often have to make decisions under conflict, for instance, to conform to the group or obey authority (subjects at higher social rank in the group). The neural mechanisms underlying how social group setting affects decision-making have largely remained unclear. In this study, we designed novel behavioral tests using food access priority and fear conditioning paradigms that captured decision-making under conflict associated with social group environments in mice and examined the roles of serotonin (5-HT) on these processes. Using these behavioral tests, administration of the selective 5-HT reuptake inhibitor, which increased 5-HT transmission, was found to attenuate conflicts in decision-making that may be associated with human cases of social obedience and conformity in mice under group housing. The results suggest that 5-HT plays important roles in the regulation of individual behaviors that organize social group dynamics.

## Introduction

Decision-making involves cognitive and affective processes in selecting actions over other available, alternative choices to achieve goals. The neural mechanisms of decision-making in relation to “neuroeconomics”, the research area that integrates economics, neuroscience, and psychology, to elucidate human decision-making processes and their deficits in psychiatric disorders, have gained increasing attention, and have been extensively investigated, over the past decade^1^. These studies have unveiled a number of key brain regions and the neurotransmitters involved in decision-making processes. In particular, neurotransmission of monoamines such as dopamine (DA) and serotonin (5-HT) is suggested to play an important role in decision-making^2,3^. The role of DA has been demonstrated primarily in non-social aspects of decision-making^4,5^, whereas several studies have shown that 5-HT is involved in social aspects of decision-making^6,7^ in humans. In accordance with the roles of monoamines on decision-making, stress, which affects DA^8^ and 5-HT^9^ transmission, has been shown to affect social decision-making, such as escaping vs. opposing the stressor known as the “fight-or-flight” response^10^, which is associated with altered neuropeptide gene expression in the amygdala^11^, as well as alterations of social discrimination and decision-making in male mice lacking corticosterone activating mineralocorticoid receptors^12^.

Decision-making is known to be influenced by social environments, such that people render decision-making under conflict, either consciously or unconsciously, depending on their presence in a social group. For instance, Milgram conducted a famous psychological study showing that people make decisions under conflict regarding obedience to authority^13^, such that hierarchy in a social group yields substantial impacts on decision-making processes. Another famous psychological study by Asch demonstrated that people in a social group also make decisions under conflict to conform to a majority of the group due to social pressure^14^. Such social conformity and obedience may not be unique phenomena in humans, and similar social group dynamics are also observed in animals. For instance, in social groups of non-human primates such as macaques, in which strict social hierarchy exists, access to rewards such as food (food access priority, or FAP) is determined based on social ranks of subjects within the group, with subjects in the dominant class accessing foods before those in the subordinate class^15^. Similar FAP in social groups are observed in various other animals, including rodents^16–19^. In addition, many animals living in groups exhibit synchronized and coordinated actions, which are often initiated by a small number of subjects and propagated into many others within the group^20,21^.

The neural mechanisms that cause conflicts associated with social conformity and obedience in decision-making remain largely unknown. Several recent studies in human subjects have attempted to unveil these neural mechanisms^22,23^. Indeed, although there have already been extensive studies using animals, including both rodents and non-human primates^24,25^, for stochastic decision-making, animal studies for social decision-making have been relatively limited. Nonetheless, some imperative animal model systems are available to examine social decision-making, such as the stress-alternatives model developed by Summers and colleagues, in which stressful social decision-making to escape or express submission against an opponent is examined in rodents^11,26^.

Accordingly, the aim of this study was two-fold. First, we developed two behavioral tests to examine behaviors similar to human social conformity and obedience (denoted as social conformity-like and obedience-like behaviors hereafter), respectively, in mice. In particular, we examined social obedience-like behavior in rodents as reward access priority that follows the hierarchical structure of social groups, whereas social conformity-like behavior was examined as propagation of a specific behavior from individuals demonstrating such behavior to those not showing it in relation to the proportion of demonstrating individuals within social groups. Then, using these behavioral tests, we examined, as a first step of unveiling the neural mechanisms of decision-making under conflict in a social group, the role of 5-HT on decision-making under conflict that may be associated with social conformity and obedience by pharmacological manipulation of 5-HT transmission with selective serotonin reuptake inhibitor (SSRI) administration. 5-HT transmission was examined in this study since it was the most promising target, based on previous studies showing that 5-HT is involved in social aspects of decision-making^6,7^.

## Results

### Decision-making for social obedience-like behavior

**Figure 1:**
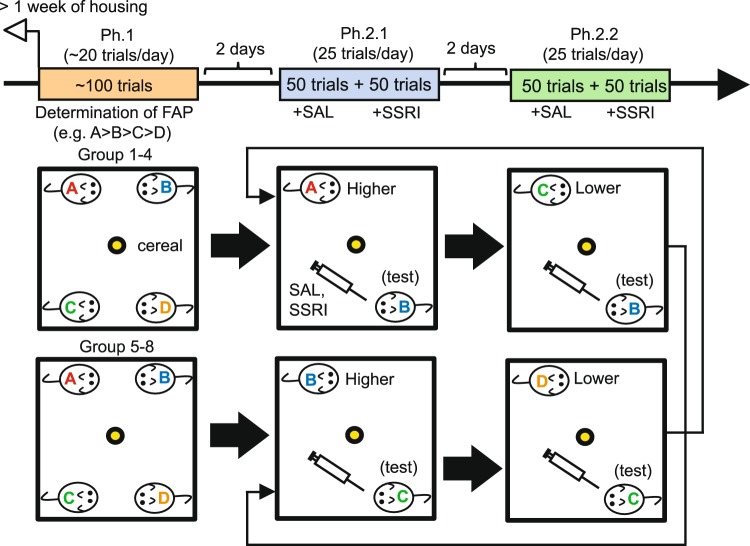
The behavioral test for decision-making under conflict associated with social obedience. Schematic diagrams illustrating the experimental design. Arrows indicate the sequence of experiments, beginning from Ph.1 to Ph.2.1 and Ph.2.2, followed by another set of Ph.2.1 and Ph.2.2.

We designed a behavioral test to investigate decision-making for social obedience-like behaviors (Fig. 1). In this test, first, we used groups of 4 mice per cage were used. The test consisted of 3 phases (Fig. 1).

**Figure 2:**
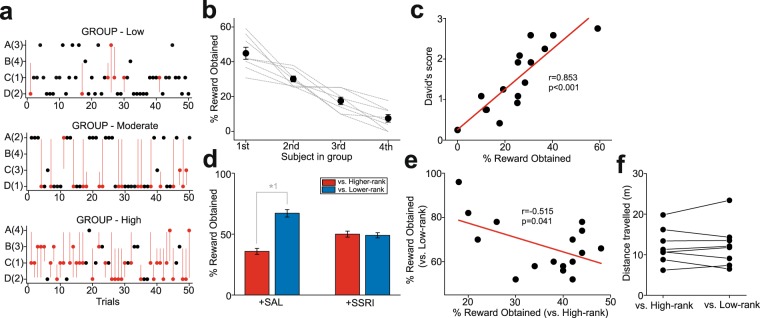
Decision-making under conflict in social obedience-like behavior and the effects of SSRI. (**a**) A graph showing 3 representative groups of trial-by-trial competition for the first 50 trials with low (top), moderate (middle), and high (bottom) frequencies of agonistic interactions between subjects during the competition. Subjects in each group are denoted as A-D, and parentheses indicate the ranks of the subjects based on the number of obtaining the rewards. Red and black circles indicate the trials with and without agonist interactions. The animals that exhibited agonist interactions were connected with red lines, and the ones that obtained the rewards were indicated with red circles. (**b**) A graph showing FAP of mice in each group (dashed lines), and the averages of all groups (circles), ranked from 1st to 4th, based on the number of first food reward access over other mice. Error bars indicate s.e.m. (**c**) A graph showing a correlation between DS and FAP. (**d**) A graph, along with illustration of individual cases, showing the number of food accesses (expressed as percentage of trials) of test subjects with SAL and SSRI administration over higher- and lower-FAP opponents. *^1^p < 0.001. (**e**) A graph showing a correlation between the number of food accesses against lower- vs. higher-FAP opponents. (**f**) A graph showing spontaneous locomotion of test subjects when they were placed with higher- or lower-FAPopponents, but without food reward presentation, in the open field chamber.

The first phase (Ph.1) was aimed at examining whether priority existed in accessing food rewards among mice within their groups, since, although FAP in social groups of some animal species such as macaques^15^ has been extensively investigated, FAP in rodent social groups has less been explored^16,19^. Thus, in Ph.1, all 4 mice from each group (8 groups, denoted as Group 1–8 in Fig. 1, n = 32) were placed in an open field chamber, and they competed for food rewards (pieces of cereal; Fig. 1). Obtaining the food rewards happened without agonistic interactions between animals in some trials, but in other trials, agonistic interactions were accompanied (Fig. 2a). Different frequencies of agonistic interactions at obtaining the rewards were observed in different groups, with some groups showing high, and other groups showing low agonistic interactions. However, when and between which animals such agonistic interactions happened were quite unpredictable in all groups (Fig. 2a). This competition unveiled a linear relationship in the order in which the mice accessed foods within each group, suggesting the presence of FAP (Fig. 2b).

Four out of 8 groups tested in Ph.1 were further subjected to a tube rank test, which is another behavioral test to examine social rank of mice, and examined its correlation between FAP. In the tube rank test, David’s score (DS), which is an index of social dominance, was calculated based on a dyadic proportion of wins and losses, as conducted in our previous studies^27,28^. A significant positive correlation was observed between FAP and DS (r = 0.853, p < 0.001; Fig. 2c), suggesting that, similar to how it operates in macaques, FAP is determined by social ranks of mice in the hierarchy.

Using the FAP paradigm, we then investigated decision-making of mice regarding whether they accessed food rewards when confronted by opponents of higher and lower social rank within the groups. Thus, in the next 2 phases (Ph.2.1 and Ph.2.2; Fig. 1), a pair of mice was selected from each group and placed in the chamber for food access competition. In Ph.2.1, 1st vs. 2nd FAP mice from Group 1–4 and 2nd vs. 3rd FAP mice from Group 5–8 were paired. The selective serotonin reuptake inhibitor (SSRI) fluoxetine (10 mg/kg, i.p.) and control treatment saline (SAL) administration were given to the lower-FAP mice in each pair (2nd in Group 1–4 and 3rd in Group 5–8; these drug-administered mice are hereafter denoted as “test subjects”) during the test. Then, in Ph.2.2, the drug-administered mice were paired with lower-FAP mice (2nd (test) vs. 3rd FAP mice from Group 1–4 and 3rd (test) vs. 4th FAP mice from Group 5–8), along with drug administration to the test subjects. Ph.2.1 and Ph2.2 were repeated with the pairing of mice reversed for Group 1–4 and Group 5–8. As expected, the test subjects with SAL administration (n = 16) exhibited a significantly lower number of food accesses when confronted by higher-FAP mice in Ph.2.1 than when confronted by lower-FAP opponents in Ph.2.2 (two-way ANOVA with repeated measures, F_1,30_ = 36.3, p < 0.001 for opponent FAP; post-hoc Tukey test, p < 0.001 in Higher vs. Lower FAP with SAL; Fig. 2d). Moreover, a negative correlation was observed between the number of food accesses in confrontation with lower- vs. higher-FAP opponents (r = −0.515, p = 0.041; Fig. 2e), suggesting that mice that were more obedient to higher-rank mates tended to behave more assertively toward lower-rank mates. We examined the effects of pharmacological manipulation of 5-HT transmission with SSRI administration (n = 16). The test subjects with SSRI exhibited an approximately equal number of food accesses against both higher- and lower-FAP opponents (F_1,30_ = 0.623, p = 0.436 in drug treatment; F_1,30_ = 40.5, p < 0.001 in interaction; p = 0.992 in Higher vs. Lower FAP with SSRI; Fig. 2d).

It is also possible that some factors other than obedience, such as anxiety, influenced behaviors of animals during the test. To examine this issue, locomotion of test subjects (n = 8) were measured for 5 minutes without food reward presentation when they were placed in the open field chamber with either higher-FAP or lower-FAP opponents. Thus, if confrontation against higher-FAP opponents heightened anxiety, it would be reflected as more freezing resulting in lower locomotion of test subjects compared to confrontation against lower-FAP opponents. However, locomotor distance of test subjects was not different between when they were placed with higher-FAP and lower-FAP opponents (paired t-test, t_7_ = 0.195, p = 0.851; Fig. 2f).

These results suggest that mice render decision-making under conflict that may be associated with obedience to authority in reward seeking when they are in social group environments, and 5-HT transmission may play a role in such decision making under conflict associated with social hierarchy.

### No relationship between non-social behavioral inhibition and social rank

**Figure 3:**
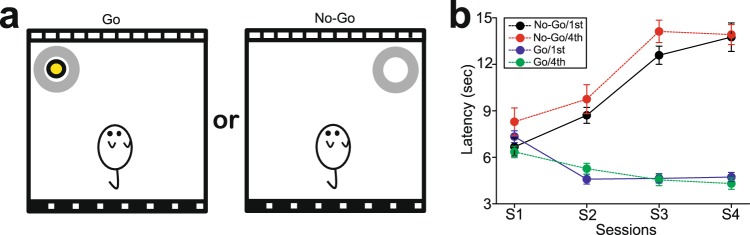
Spatial Go/No-go test in mice at higher and lower social ranks. (**a**) Schematic diagrams illustrating Go (a bowl with a food reward) and No-go (a bowl only) trials of the spatial Go/No-Go test. Spatial cues are provided on the walls of the chamber. (**b**) A graph showing latencies to access the bowl in Go and No-go trials over 4 sessions in mice at the highest (1st) and lowest (4th) FAP in their social groups.

The findings in the social obedience test suggest that mice at lower FAP should have stronger behavioral inhibition than those at higher FAP, as lower-FAP mice are required to inhibit their food access more frequently than higher-FAP mice. To investigate this issue, we conducted the spatial Go/No-go test, which is similar to the test used by Dumont and colleagues^29^ (Fig. 3a). In this test, a bowl with (Go trial) or without (No-go trial) a food reward (a piece of cereal) was presented in a specific location within the open field chamber, with spatial cues provided on the walls in each trial. Thus, animals could judge whether there was a reward within the bowl by the location of the bowl.

Mice at the 1st (highest, n = 4) and 4th (lowest, n = 4) FAP in each group quickly learned the presence or absence of rewards within the bowl, such that the latency to reach the bowl with rewards was significantly decreased (two-way ANOVA with repeated measures for Go trials, F_3,568_ = 20.8, p < 0.001 for session), whereas the latency to reach the bowl without rewards was significantly increased (two-way ANOVA with repeated measures for No-go trials, F_3,184_ = 38.4 p < 0.001 for session) over the sessions (Fig. 3b). However, no difference was observed in performance of both Go (F_1,568_ = 0.742, p = 0.391 for social rank; F_3,568_ = 2.01, p = 0.112 for interaction) and No-Go (F_1,184_ = 0.231, p = 0.632 for social rank; F_3,184_ = 1.92, p = 0.130 for interaction) trials between 1st- and 4th-FAP mice (Fig. 3b).

These results suggest that inhibition on food access exhibited by test subjects when they were against higher-FAP mice may not be because of difference in the ability of behavioral inhibition, but may be associated with social stress (stress given by higher-FAP mice onto lower-FAP mice).

### Decision-making for social conformity-like behavior

**Figure 4:**
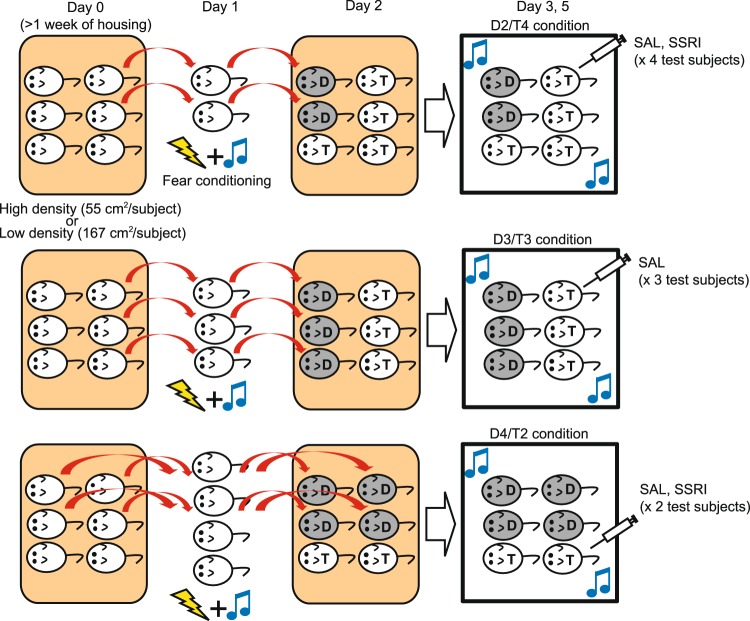
The behavioral test for decision-making under conflict associated with social conformity. Schematic diagrams illustrating the experimental design with different (D2/T4, D3/T3, and D4/T2) group conditions.

**Figure 5:**
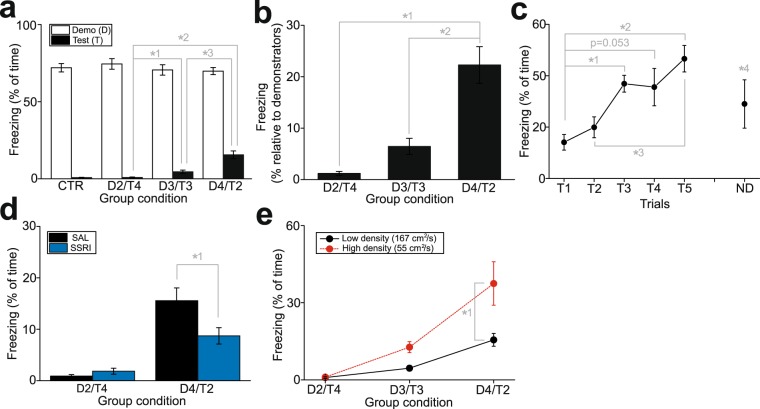
Decision-making under conflict in social conformity-like behavior and the effects of SSRI. (**a**) A graph showing freezing of demonstrators (D) and test subjects (T) with SAL in different group conditions. Control groups in which all 6 mice were subjected for fear conditioning or none of them were conditioned is denoted as CTR. *^1–3^p < 0.001. (**b**) A graph similar to (**a**), but showing as percentages that the test subjects expressed freezing relative to the demonstrators in different group conditions. *^1–2^p < 0.001. (**c**) A graph showing growing freezing of test subjects in the D4/T2 conditions with repeated trials (T1-T5), and one additional trial, but without demonstrators in the cage (ND; 6th trial). *^1^p = 0.037, *^2^p = 0.002, *^3^p = 0.012, *^4^p = 0.017. (**d**) A graph showing freezing of test subjects with SAL (data shown in (**a**)) and SSRI administration. *^1^p = 0.020. (**e**) A graph showing freezing of test subjects housed in smaller (55 cm^2^/subject; high housing density) and larger (167 cm^2^/subject; low housing density; data shown in (**a**)) cages. *^1^p < 0.001.

We also designed another behavioral test to investigate decision-making under conflict for social conformity-like behavior (Fig. 4). In this test, groups of mice, housed 6 mice per cage, were used (at the housing density of 167 cm^2^/subject). First, either 2, 3, or 4 mice randomly selected from each group were subjected to cued fear conditioning by auditory tone and foot shock (these mice are hereafter denoted as “demonstrators”), then returned to their home cages for housing with the remaining non-conditioned mice (these mice are hereafter denoted as “test subjects”). Accordingly, there were 3 different group conditions; D2/T4 (2 demonstrators and 4 test subjects per group; n = 12 test subject, n = 6 demonstrators; 3 groups), D3/T3 (3 demonstrators and 3 test subjects per group; n = 9 test subjects, n = 9 demonstrators; 3 groups), and D4/T2 (4 demonstrators and 2 test subjects per group; n = 10 test subjects, n = 20 demonstrators; 5 groups). In addition, as controls, one group with all 6 mice subjected for fear conditioning, and another group with none of mice subjected to fear conditioning, were examined. One day after housing demonstrators and test subjects together, these mice were placed in the open field chamber, and the auditory tone used for fear conditioning was presented to assess their freezing behaviors during the tone. Demonstrators in the D2/T4, D3/T3, and D4/T2 conditions exhibited substantial freezing during the tone but without significant difference between the conditions (one-way ANOVA, F_3,37_ = 0.394, p = 0.758; Fig. 5a). In contrast, although test subjects in the D2/T4 condition did not exhibit freezing, those in the D3/T3 and D4/T2 conditions exhibited significant freezing during the tone, and the rate of freezing increased as the number of demonstrators increased from the D3/T3 to the D4/T2 conditions (F_3,33_ = 23.5, p < 0.001; Fig. 5a). Fig. 5b also shows freezing of test subjects, but expressed as percentages relative to the averages of freezing of demonstrators in each condition, illustrating that the amount of freezing in test subjects was up to approximately 25% of freezing of demonstrators in the D4/T2 condition (one-way ANOVA, F_2,28_ = 26.5, p < 0.001).

We further examined whether freezing of test subjects depended on social learning from demonstrators. Three groups in the D4/T2 conditions received repeated auditory tone presentations, and the amount of freezing in test subjects was measured. The auditory cue was presented once per day for 6 days. The amount of freezing in the test subjects was significantly increased as more number of trials were given to the 5th trial (one-way ANOVA with repeated measures, F_4,20_ = 6.72, p = 0.001; Fig. 5c). On the 6th auditory tone presentation, demonstrators were removed from the cage, and only test subjects remained. In this condition, test subjects exhibited substantially lower amount of freezing compared to the 5th trial, but was still comparable to the 1st trial, and significantly higher than the control group (unpaired t-test, t_10_ = 2.86, p = 0.017 compared to CTR shown in Fig. 5c).

Upon the findings of the effects of SSRI on decision-making associated with social obedience, we also examined the effects of SSRI administration on freezing of test subjects. In the D2/T4 condition (n = 12 test subjects, n = 6 demonstrators; 3 groups), freezing of test subjects with SSRI administration was no different from those with SAL administration, whereas in the D4/T2 condition (n = 10 test subjects, n = 20 demonstrators; 5 groups), SSRI administration significantly decreased freezing during the tone compared to those with SAL administration (two-way ANOVA with repeated measures, F_1,34_ = 3.90, p = 0.057 for drug treatment; F_1,34_ = 39.7, p < 0.001 for group condition; F_1,34_ = 4.60, p = 0.039 for interaction; Fig. 5d).

We also found that housing density affected freezing of test subjects. Compared to test subjects in the groups housed in larger cages (167 cm^2^/subject; low housing density), test subjects in the groups housed in smaller cages (55 cm^2^/subject; high housing density; 3 groups each in D2/T4 [n = 12 test subjects, n = 6 demonstrators], D3/T3 [n = 9 test subjects, n = 9 demonstrators], and D4/T2 [n = 10 test subjects, n = 20 demonstrators] conditions) exhibited increased freezing in the D4/T2 condition (two-way ANOVA, F_1,52_ = 23.0, p < 0.001 in housing density; F_2,52_ = 50.0, p < 0.001 in group condition; F_2,52_ = 8.99, p < 0.001 in interaction; Fig. 5e).

These results suggest that rodents in social groups exhibit behaviors similar to human social conformity, which may involve 5-HT transmission.

## Discussion

In this study, we have developed behavioral tests in rodents that enable us to investigate decision-making under conflict in social group conditions and have shown that 5-HT transmission plays an important role in decision-making under conflict in causing social obedience-like and conformity-like behaviors.

We examined social obedience-like behavior using the FAP paradigm. In social groups of non-human primates such as macaques, as well as other wild animals, linear social hierarchy exists, and a correlation between social ranks and FAP has been reported^15,30^. In contrast, although presence of a linear social hierarchy in rodent social groups has been observed^27,28,31^, whether mice living in social groups also exhibit FAP had yet to be determined. In this study, we found that, similar to non-human primates, FAP correlated with social ranks is also present in mouse social groups. Although motivation for food rewards could affect the order of animals to access foods, a contribution of such a factor to the current observation would not be significant, since only difference in the paired testing condition was whether test subjects confronted against higher or lower rank opponents. In particular, some mice that were more obedient to higher-rank mates behaved in a more dominant way toward lower-rank mates in food access competitions, suggesting that these mice were more strictly following the hierarchy. In contrast, increasing 5-HT transmission with SSRI administration resulted in less clear FAP and conflicts in decision-making for food access. This finding is consistent with studies in non-human primates^32^ and reptiles^33^ showing that SSRI administration causes more elastic social hierarchy, with not only the attenuation of submissive behaviors in low social rank subjects but also the concurrent attenuation of dominant behaviors in high social rank subjects. Studies have shown that several individual behavioral characteristics (e.g. aggression, behavioral inhibition) are important factors for determining social hierarchy^34^. Thus, it would be possible that animals with stronger behavioral inhibition tend to be at lower social rank than those with weaker inhibition, since lower social rank subjects are required to inhibit their food access more frequently than those at higher social ranks, since mice at lower FAP were expected to show stronger behavioral inhibition than those at higher FAP. If this is the case, regardless of whether there is stress (of any kind, including social stress) or not, lower rank animals exhibit stronger behavioral inhibition than mice at higher rank even in non-social contexts. In contrast to this expectation, however, we found that behavioral inhibition assessed in the spatial Go/No-go test did not differ between higher- and lower-FAP subjects, suggesting that the ability of behavioral inhibition that each mouse has may not be a major factor involved in social obedience-like behaviors observed in the test, and social stress in lower rank mice given by higher rank mice induces such social obedience-like behaviors. Another possible explanation for such a discrepancy may be that neural mechanisms that mediate non-social domains of behavioral inhibition may also be different from those involved in social domains of behavioral inhibition, although some aspects of mechanisms such as 5-HT may still mutually be involved in both social and non-social domains^35^.

Social conformity-like behavior in rodents was investigated using the fear conditioning paradigm. Emotional contagion or social transmission of information has been reported to take place in rodents^36,37^. An elegant series of studies by Monfils and colleagues have shown that animals that are not fear conditioned, but housed with those with fear conditioning, start to exhibit significant freezing in response to the tone used in fear conditioning^38–40^. In these studies, non-conditioned rats paired with conditioned rats (1 conditioned and 1 unconditioned rats in one cage) exhibited freezing for approximately 10–20% of time during the tone. In our study, percentages of freezing were increased with increasing the number of demonstrators, and this 10–20% of time of freezing was observed only when the number of demonstrators exceeded the number of test subjects, i.e. the D4/T2 condition. Therefore, these observations at least partly comply with the fact that emotional contagion may play a significant role in freezing of test subjects in the behavioral paradigm developed in this study. Test subjects were also initially found to exhibit freezing at a rate less than 25% of the demonstrators even in the D4/T2 condition, which would be too weak to refer this observation as social conformity reported in human social psychological studies. Thus, instead of social conformity, this observation appeared to be more appropriate to interpret as a kind of behavioral mimicking plasticity. Such mimicking may involve observational learning, and a recent study has reported that mice learn to exhibit freezing by observation of other mice demonstrating freezing behavior^41^. Based on the study suggesting that social conformity involves social learning^42^, we further examined whether the amount of freezing changed across repeated auditory tone presentations. In the D4/T2 condition, we observed that the amount of freezing in test mice was not substantially higher compared to demonstrators at the first time of tone presentation, but it has increased over several times of repeated presentations of the auditory cue. Moreover, after such repeated trials, moderate, but significant, freezing could still be induced by the auditory tone in test subjects even when demonstrators were removed from the groups, suggesting that mimicking may not be a whole factor that causes freezing in response to the auditory tone. Collectively, emotional contagion, mimicking, and social (observational) learning may be intermingled factors that are involved in causing freezing behavior of test subjects. In this study, we also found that housing density yielded substantial impacts on social conformity-like behavior, although it remains unclear whether this effect was due to housing density itself or stress associated with social crowding.

In this study, we examined the effects of pharmacological manipulation of 5-HT transmission, as this neurotransmitter, along with several other neural factors such as stress and anxiety^11,26^, has previously been shown to be involved in social aspects of decision-making^6,7^. Indeed, it is important to note that the effects engendered by SSRI treatments does not necessarily lead to the conclusion that 5-HT is naturally involved in the observations reported in this study. For instance, 5-HT and DA have extensive interaction with each other, and alterations in one of them consequently alter the other molecule^43^. Thus, it is highly unlikely that 5-HT is the only neurotransmitter involved in decision-making under conflict in social group environments. We have previously shown that DA, through activation of D1 and D2 receptors, plays an important role in the construction of social hierarchy in both rodents and primates^27,28^, such that DA is also likely to affect decision-making under conflict associated with social group setting, although DA may mediate different aspects of the decision-making process than 5-HT. It is also important to examine whether activation of fear conditioning-associated limbic regions such as the amygdala^44^ can be observed in test subjects under the social conformity test, since, if freezing of test subjects is due to emotional contagion, such limbic areas may be activated, whereas if freezing is caused by social pressure, other brain areas may be primarily activated.

Similar to DA^45^, 5-HT transmission also consists of tonic, background extracellular tone and phasic, synaptic release^46,47^. Since SSRI inhibits 5-HT reuptake, its administration is thought to increase synaptic 5-HT release^48^. Nonetheless, the effects of acute SSRI administration on brain functionsin rodents have been reported to yield an inverted U-shape relationship, through 5-HT_2A_ receptor^49,50^. Indeed, such inverted U-shape relationship has also been reported in DA functions^51^. These observations suggest that low 5-HT signal (low 5-HT transmission) would be amplified, whereas high 5-HT signal (high 5-HT transmission) would be attenuated, by SSRI administration, which may explain the opposite effects of SSRI in high and low social rank mice, such that mice at higher and lower rank attenuated dominance and submission, respectively, with SSRI treatments. SSRI administration also elevates extracellular tonic 5-HT tone^52^, which may facilitate tonic, constitutive 5-HT_1A_ receptor stimulation^53,54^. Such tonic 5-HT alterations may contribute more on social conformity, since no observation of the inverted U-shape relationship of the SSRI effects on social conformity, e.g. facilitation of conformity in low number of demonstrators, whereas attenuation of conformity in high number of demonstrators with SSRI administration. Although speculative, decision-making associated with social conformity and obedience may involve distinct patterns of 5-HT transmission, with tonic extracellular stimulation of 5-HT_1A_ receptor and phasic synaptic stimulation of 5-HT_2A_ receptor, respectively. A further study with selective pharmacological manipulations of 5-HT_1A_ and 5-HT_2A_ receptor could address this issue.

In conclusion, our study reported that 5-HT may be one of the important neural substrates involved in decision-making under conflict of individuals that are organized within social groups. Since this study is an initial step of the investigation, many questions remain to be addressed in future studies, such as whether other neurotransmitters such as DA are also involved, whether and how these observations are similar or different across different strains of mice and different species of animals, and which brain areas and neural circuits are involved.

## Methods

### Animals

Adult male ICR mice (Crl:CD1(ICR), 8 weeks old at arrival) were purchased from Charles-River Japan. These mice were grouped into 4 or 6 subjects per cageand housed for at least 1 week, but no longer than 1 month, before starting experiments. They remained housed together until all experiments were completed. Animals were housed in a temperature-controlled room with a normal light-dark cycle. Food and water were unrestricted throughout the experiments. All experiments were conducted in accordancewith the *Guidelines for Proper Conduct of Animal Experiments by the Science Council of Japan* and were approved by the Kyoto University Primate Research Institute animal ethics committee.

### Drug preparation and administration

The selective serotonin reuptake inhibitor (SSRI) fluoxetine was purchased from Wako Pure Chemical Industries, Ltd. A fluoxetine dose of 10 mg/kg was dissolved in 0.2 ml of 0.9% saline and administered intraperitoneally (i.p.) approximately 10 minutes before tests. This dose of fluoxetine was selected due to reports from previous studies finding that fluoxetine administration at this dose causes several behavioral changes in rodents^55,56^. As a control treatment, the equivalent volume of saline (SAL) was given to animals.

### Social obedience test

In this test, cereals were given as food rewards. All animals had experienced consuming cereals at least once prior to experiments in their home cages under the isolated condition (other cage mates were temporarily removed from the home cages while cereals were given to a specific animal), to exclude a possibility of neophobia to the cereals affecting the test. An experimental design of the social obedience test is illustrated in Fig. 1. The social obedience test utilizing the food access priority (FAP) paradigm was conducted using groups of mice that were housed 4 mice per cage. The test consisted of 3 phases. In the first phase (Ph.1), all 4 mice in each group were placed in the open field chamber (40 × 40 × 40 cm) located in the dark room with a dim light and habituated for 10 minutes before beginning the session on each day. After the habituation period, a piece of cereal (a reward) was placed in the chamber at an approximately equal distance from all mice and left until it was accessed and consumed by any of the mice, or until 60 seconds had passed without access, whichever came first. This process was repeated for up to 20 trials per session per day. The mouse reaching the reward first in each trial was recorded. Ph.1 ended when we reached 105–110 trials. This Ph.1 determined the FAP of mice in each group (e.g., mouse A is the highest and mouse D is the lowest FAP in Fig. 1). Two days after completion of Ph.1, Ph.2.1 and Ph.2.2 were conducted sequentially, with an interval of 2 days between the phases. In Ph.2.1 and Ph.2.2, a pair of mice was selected from each group and placed in the chamber. In Ph.2.1, a pair selected from Groups 1-4 involved 1st (mouse A in Fig. 1) vs. 2nd (mouse B) FAP, and a pair selected from Groups 5-8 involved 2nd (mouse B) vs. 3rd (mouse C) FAP. In Ph.2.2, a pair selected from Groups 1-4 involved 2nd (mouse B) vs. 3rd (mouse C) FAP, and a pair selected from Groups 5-8 involved 3rd (mouse C) vs. 4th (mouse D) FAP. Ph.2.1 and Ph.2.2 consisted of 100 trials each, with 25 trials per session per day. These 100 trials in each phase were further divided into 50 trials each for SAL and SSRI administration, respectively. The test mice (mice B and C from Group 1-4 and Group 5-8, respectively) received the drug treatments. These drug treatments were given to the test mice once per day, 10 minutes before starting the session each day. After completion of a set of Ph.2.1 and Ph.2.2, another set of Ph.2.1 and Ph.2.2 was conducted, along with conversion of the pairs between Groups 1-4 and Groups 5-8. In other words, in Ph.2.1, a pair from Groups 1-4 was selected as 2nd vs. 3rd FAP, and that from Groups 5-8 was selected as 1st vs. 2nd FAP, whereas in Ph.2.2, a pair from Groups 1-4 was selected as 3rd vs. 4th FAP, and the pair from Groups 5-8 was selected as 2nd vs. 3rd FAP. Drug treatments were given to the 3rd mice from Groups 1-4 and the 2nd mice from Groups 5-8.

### Tube rank test

Social ranks of mice in each group used in the social obedience test was examined using a tube rank test, as conducted in our previous studies^27,28^. Briefly, a pair of mice from each group, housed at 4 mice per cage, was placed near a 30 cm long × 2.8 cm diameter tube, with one mouse at each end. When the mouse from each side reached the middle of the tube, the partition wall was removed. The mouse that forced the other to retreat was designated as the “winner”, and the mouse that retreated from the tube was designated as the “loser”. A win and loss were scored as +1 and 0, respectively. Tournaments of all possible combinations of matches by pairs of mice in each group were conducted 5 times. Then, social dominance was quantified by David’s score (DS)^27,57^, with higher DS indicating greater dominance.

### Spatial Go/No-go test

A non-social domain of behavioral inhibition was investigated using the spatial Go/No-go test, which is similar to the test used in the study by Dumont and colleagues^29^ (Fig. 3a). In this test, a bowl (3.5 cm diameter) filled with sawdust was presented at specific locations within the open field chamber (e.g., upper left and right corners of the chamber in Fig. 3a), with spatial cues provided on the walls. When the bowl was presented in one place, a piece of cereal (a reward) was embedded in the bowl, whereas the bowl presented in the other place was empty. A trial began with the introduction of a mouse into the chamber. The mice subjected to the test were at the highest and lowest FAP in each group used in the social obedience test. The mouse was allowed free access to the bowl for 20 seconds in each trial. A trial ended either when the mouse accessed the bowl or 20 seconds had passed, whichever came first. The latency thata mouse accessed the bowl was measured in each trial. One session consisted of 24 trials, and 4 sessions (S1-S4) were given for each mouse, with one session per day. After the trial, the mouse was returned to the home cage until the next trial. Inter-trial intervals were approximately 30 seconds. Presentations of the bowl with rewards (Go trial) and without rewards (No-go trial) were given pseudo-randomly at the ratio of 3:1 (18 Go trials and 6 No-go trials in one session).

### Social conformity test

An experimental design of a social conformity test is illustrated in Fig. 4. A social conformity test utilizing the cued fear conditioning paradigm was conducted using 23 groups of mice that were housed 6 per cage (n = 138 mice in total). These mice were housed in either large (n = 11 groups, 167 cm^2^/subject; 25 × 40 cm; 1,000 cm^2^) or small (n = 9 groups, 55 cm^2^/subject; 15 × 22 cm; 330 cm^2^) cages. On the first day of the test, either 2, 3, or 4 mice randomly selected from each group were subjected to cued fear conditioning. Cued fear conditioning was conducted using a custom-madetransparent acrylic chamber (20 × 20 × 20 cm) with a metal grid floor, which was connected to the pulse generator and stimulus isolator. After leaving mice for 10 minutes in the chamber for habituation, an auditory tone (5 kHz, 80 dB) was given for 20 seconds. During the last 1 second of the tone, an electrical foot shock at 0.6 mA was given. This process was repeated 3 times, with 2 minutes of inter-trial intervals. Approximately 30 seconds after the last foot shock, the conditioned mice were returned to their home cages and housed together with other non-conditioned mice. These mice subjected to fear conditioning were denoted as demonstrators (D), whereas other non-conditioned mice remaining in the groups were denoted as test subjects (T). Accordingly, depending on the number of fear-conditioned subjects, groups were divided into 3 conditions: D2/T4 (i.e., 2 mice receiving fear conditioning, n = 3 for each of the large and small cages), D3/T3 (i.e., 3 mice receiving fear conditioning, n = 3 for each of the large and small cages), and D4/T2 (i.e., 4 mice receiving fear conditioning, n = 5 and n = 3 for the large and small cages, respectively). In addition, as controls, one group with all 6 mice subjected to fear conditioning, and another group with none of mice subjected to fear conditioning, were examined. Demonstrators and test subjects were housed together for an additional 24 hours in the same cages on the second day of the test. On the third day of the test, test subjects in some groups received SAL, and test subjects in other groups received SSRI administration (drug treatments of the same type within each group). Then, all 6 mice with a mixture of demonstrators and test subjects in each group were placed in the open field chamber. Ten minutes after a habituation period, an auditory tone that was identical to the one used in fear conditioning was given for 20 seconds, and movements of mice were video-recorded during the tone presentation at the rate of 30 fps sampling. Two days after this trial (on the fifth day of the test), another trial was conducted in which test subjects in the groups with SAL treatments on the third day now received SSRI, and vice versa. The effects of SSRI administration were examined only in the large-cage groups at the D2/T4 and D4/T2 conditions. Freezing of mice was analyzed later off-line. Using the MTrackJ plug-in of the ImageJ software^58^, which enabled manually tracking moving objects through frame-by-frame advance of video recordings, the heads of mice were tracked for 20 seconds of video recording. Freezing was defined as immobility of their heads, i.e., heads moved less than 0.5 cm for 100 ms (3 frames of recordings) or longer. This criterion was used, since, when percentages of freezing in some demonstrators were also examined with different criteria (200 ms, or 6 frames; 133 ms, or 4 frames; and 66 ms, or 2 frames of video recordings at 30 fps) and compared to that with 100 ms criterion, the differences of percentages between 100 and 133 ms criteria as well as between 100 and 200 ms criteria were less than 2% (on average, percentages of freezing were 0.89 ± 0.29% and 1.81 ± 0.60% shorter with 133 and 200 ms criteria, respectively, than that with 100 ms criterion). In contrast, when 66 ms criterion was used, the difference of percentage was almost 10% (on average, percentage of freezing was 8.56 ± 1.60% longer with 66 ms criterion than that with 100 ms criterion).

### Statistical analysis

Investigators who were not blinded to the experimental conditions conducted data collection and statistical analyses. Sample sizes were not statistically predetermined. All statistical analyses were conducted using Statistica and Origin Pro software. A probability value of p < 0.05 was considered as statistical significance. In the social obedience test, two-way analysis of variance (ANOVA) with repeated measures was used for statistical analysis, with drug treatments (SSRI vs. SAL) as a within-subject factor and FAP of opponents (higher vs. lower FAP than the test subjects) as a between-subject factor. Similarly, in the spatial Go/No-go test, two-way ANOVA with repeated measures was used for each of Go and No-go trials, with sessions (S1–S4) as a within-subject factor and FAP (1st vs. 4th FAP in the groups) as a between-subject factor. In the social conformity test, one-way ANOVA and two-way ANOVA with or without repeated measures were used depending on the comparisons, with test conditions (D2/T4 vs. D3/T3 vs. D4/T2) and housing density (167 cm^2^/subject vs. 55 cm^2^/subject) as between-subject factors, and drug treatments (SAL vs. SSRI) as a within-subject factor. Post-hoc analyses were conducted using the Tukey test.

### Data availability

The datasets generated and analyzed during the current study are available from the corresponding author upon reasonable request.
